# Malaria Mosquito Host-Seeking Activity Times in Manhiça District, Rural Mozambique, and the Need to Better Match Entomological Surveillance Strategies to Daylight Cycles

**DOI:** 10.3390/insects16121264

**Published:** 2025-12-12

**Authors:** Ndey Bassin Jobe, Mara Máquina, Mercy A. Opiyo, Helena Martí-Soler, Arlindo Malheia, Dulcisária Marrenjo, Nelson Cuamba, David Pino, Francisco Saúte, Krijn P. Paaijmans

**Affiliations:** 1 Center for Evolution and Medicine, School of Life Sciences, Arizona State University, Tempe, AZ 85281, USA; 2 Centro de Investigação em Saúde de Manhiça (CISM), Fundação Manhiça, Maputo 1929, Mozambique; mara.maquina@manhica.net (M.M.);; 3 ISGlobal, 08036 Barcelona, Catalonia, Spain; 4 Malaria Elimination Initiative, University of California San Francisco, San Francisco, CA 94143, USA; 5 Programa Nacional de Controlo da Malária, Ministério da Saúde, Maputo 264, Mozambique; 6 PMI VectorLink Project, Abt Associates Inc., Maputo 1102, Mozambique; 7 Department of Physics, Universitat Politècnica de Catalunya·BarcelonaTech, 08034 Barcelona, Catalonia, Spain; david.pino@upc.edu; 8 Institute of Space Studies of Catalonia, 08860 Castelldefels, Catalonia, Spain; 9 Simon A. Levin Mathematical, Computational and Modeling Sciences Center, Arizona State University, Tempe, AZ 85281, USA; 10 WITS Research Institute for Malaria (WRIM), Faculty of Health Sciences, University of the Witwatersrand, Johannesburg 2050, South Africa

**Keywords:** *Anopheles funestus*, *Anopheles ziemanni*, *Anopheles gambiae*, *Anopheles squamosus*, dawn, dusk, diurnal activity

## Abstract

Daytime biting malaria mosquitoes present a challenge for malaria elimination efforts in sub-Saharan Africa. Diurnal mosquito host-seeking activity times were assessed indoors and outdoors in two villages in the Manhiça district. Subsequently, sunrise and sunset data across longitudes and latitudes spanning the African continent were used to assess if current entomological surveillance approaches align with (seasonal changes in) local daylight cycles. The most abundant species was *Anopheles tenebrosus*, and daytime (defined as the period between 06:00 and 18:00) host-seeking was only observed in a single *Anopheles ziemanni*. However, sunset occurred at 17:06 on that day, which means this specimen (collected between 16:00 and 18:00) could have been collected during nighttime. Further analysis of sunrise and sunset times across several African countries show these can change by over 2 h throughout the year, depending on longitude and latitude. As changes in daylight cycles can affect mosquito and human behaviors (e.g., time spent indoors and sleeping), surveillance strategies need to be adjusted to match actual daylight cycles and/or important human behavioral components. This will allow us to collect more actionable data to inform malaria control and elimination programs.

## Simple Summary

Daytime biting malaria mosquitoes present a challenge for malaria elimination efforts in sub-Saharan Africa. We report diurnal mosquito host-seeking activity times in two villages in the Manhiça district, southern Mozambique. We also report sunrise and sunset data across longitudes and latitudes spanning the African continent, and discuss how the use of daylight cycle information can lead to the collection of more actionable data and better inform malaria control and elimination programs.

## 1. Introduction

Malaria remains one of the deadliest mosquito-borne diseases in the world. In 2023, approximately 263 million cases were reported (94% in the World Health Organization’s (WHO) African region) and the disease claimed the lives of approx. 597,000 people (95% in the WHO African region) [1]. Five countries—Nigeria (26%), the Democratic Republic of the Congo (13%), Uganda (5%), Ethiopia (4%) and Mozambique (4%)—accounted for more than half of all cases globally in 2023 [1]. Focusing on Mozambique, local malaria elimination in its southern parts (mainly Maputo Province) has been on the agenda since 1999 when the Lubombo Spatial Development Initiative (LSDI) was established. This was later followed by the MOSASWA (Mozambique, South Africa and Swaziland) initiative [2]. These cross-border initiatives aim to achieve malaria elimination in Mozambican’s neighboring countries Eswatini and South Africa, and to move from control to pre-elimination in southern Mozambique. Reducing malaria in southern Mozambique is critical, as most malaria cases in South Africa and Eswatini are imported from Mozambique [3,4,5].

The frontline malaria control tools used in the area have been (i) insecticides that target the mosquito vector via indoor residual spraying (IRS) [2,6] and/or insecticide-treated nets (ITNs) [7], (ii) diagnostic tools such as rapid diagnostic tests (RDTs) to detect the malaria parasite [8] and (iii) the prescription of malaria drugs (artemisinin-based combination therapy) [2,6]. LSDI and MOSASWA have been successful to a certain extent. For example, the LSDI resulted in an 85% reduction in malaria cases in Mozambique in 2011 [9]. However, the initial goal of MOSASWA (zero local transmission in Swaziland, South Africa and Maputo Province (Mozambique) by 2020) was not yet met [2].

Between 2015 and 2018, the Magude project was piloted to assess the viability of eliminating malaria locally in a single district (Magude district, Maputo Province) using mass drug administration (MDA) in addition to the aforementioned interventions [10]. While reductions in the prevalence (84.7%) and incidence (65.6%) of malaria cases were reported, local malaria elimination was not achieved [10]. Some of the observed gaps in protection that led to persistent local malaria transmission included (i) challenges in the coverage and adherence of mass drug administration (MDA) [10], (ii) low access and use of LLINs across years and seasons [11], and (iii) lack of public trust for IRS acceptability demonstrated through, e.g., wall modifications (re-plastering and/or washing) after IRS coverage [12], and (iv) IRS residual efficacy and pace of spraying [13].

Apart from those human-related factors, several mosquito-related factors may explain persistent malaria, including (i) the growing resistance in anopheline mosquitoes to the available public health insecticides, and to pyrethroids in particular, which are mainly used in LLINs [14,15,16,17], (ii) vector species abundance and diversity [18], and (iii) a significant portion of the mosquito population being active before community members go to bed and use bed-nets [11].

Recently, daytime biting behaviors of malaria vectors has been highlighted as a challenge for residual malaria transmission and malaria elimination efforts in, e.g., the Central African Republic [19], Senegal [20] and Kenya [21]. This is largely because people are unprotected from host-seeking malaria vectors during daytime hours. Although these studies demonstrate that daytime or late-morning biting can occur, none to our knowledge have explicitly examined how host-seeking activity aligns with actual sunrise and sunset times or seasonal changes in daylight cycles. This distinction is important because interpreting biting behavior without reference to real-time daylight patterns may mask crepuscular or diurnal activity. Our work therefore adds a complementary perspective by linking mosquito host-seeking behavior to measured solar-cycle data. This phenomenon was not studied during the Magude project, where entomological surveillance was conducted from 18:00 until 6:00 the following morning [11,18], in line with most malaria mosquito entomological surveillance programs that are conducted between sunset (often starting at 18:00 or 19:00) and sunrise (often ending at 06:00 or 07:00) (e.g., [22,23,24,25]). Although this nocturnal surveillance window is standard practice, the rationale is often based more on long-standing assumptions than on explicit empirical evidence. Historically, *Anopheles* species have been characterized as predominantly nighttime feeders, a view that has shaped surveillance protocols for decades. However, detailed evaluations of biting behavior outside the conventional nighttime window remain surprisingly limited, and these assumptions may have contributed to the under-recognition of crepuscular or even diurnal host-seeking activity. This gap highlights the need for more comprehensive temporal sampling, particularly in settings undergoing major vector control interventions that may shift biting patterns. To investigate if daytime biting malaria mosquitoes may be one of the explanatory factors of persistent malaria transmission in southern Mozambique, a pilot study was conducted to assess the indoor and outdoor host-seeking behaviors of local anopheline mosquitoes at bi-hourly intervals in the Manhiça district. Additionally, considering that sunset and sunrise times vary throughout the year in a single location and across different latitudes and longitudes, we discuss the implications of these variations for optimizing entomological surveillance strategies.

## 2. Materials and Methods

### 2.1. Study Area

The preliminary entomological study was conducted in two villages, Palmeira and Ribangua, located in the Manhiça district of Maputo province (Figure S1). The region experiences persistent malaria transmission, despite prompt diagnosis and effective treatment of confirmed malaria cases, IRS and LLINs [2,10]. The two villages are situated adjacent to large-scale sugar cane plantations, and the local communities are primarily engaged in farming activities [26]. The average annual temperature is about 23 °C, but there are two distinct seasons: the warm/rainy season from November to April (with the highest mean temperature of approx. 27 °C in January) and the cool/dry season from May to October (with the lowest mean temperatures in June–July of approx. 19 °C) [27].

### 2.2. Mosquito Surveillance

Indoor and outdoor host-seeking behaviors of *Anopheles* mosquitoes were quantified from September 2020 to July 2021 in Ribangua, and from March to July 2021 in Palmeira. The first selected house was typically the home of the bairro leader; the other houses were selected as follows: following the roads in all directions from the first house, every 3rd household was visited. If all inclusion criteria were fulfilled (i.e., homeowner present, homeowner agrees to participate, adequate and safe space for placing the tent trap indoors and outdoors, and adult male volunteer present to sleep in the tent trap), the household was enrolled in the study. If not, the neighboring household was visited. Mosquitoes were collected using human-baited tent traps (or HBTTs, see [28]). In summary, this method consists of a tent (Natural Instincts Highveld 3; L × W × H: 2.1 × 2.1 × 1.3 m) that is equipped with a CDC Miniature Light Trap (CDC-LT; Model 512, John W. Hock, Gainesville, FL, USA) that is placed on top of a Collection Bottle Rotator (Model 1512, John W. Hock, USA) between the inner and outer tent. An adult volunteer was present inside to lure mosquitoes, while being protected from mosquito bites by the inner tent.

Mosquitoes were collected during 2 h intervals for a period of 12 h in one of the following environments: (i) indoors during daytime (06:00–18:00), (ii) outdoors during daytime, (iii) indoors during nighttime (18:00–06:00), and (iv) outdoors during nighttime. The volunteer was an adult member of the household (usually the head of the household) where the tent was positioned. A fieldwork supervisor ensured the volunteers were present in the tent during trapping periods, and that all equipment functioned properly. *Anopheles* mosquitoes were identified to species or species groups using a stereomicroscope and dichotomous key of Gillies and Coetzee [29].

A total of 24 houses (16 houses in Palmeira and 8 houses in Ribangua) were selected for this study. Each week, eight houses were sampled in Palmeira and four houses in Ribangua. The same households were typically sampled for both daytime biting mosquitoes (06:00–18:00) followed by nighttime biting mosquitoes (18:00–06:00), before moving to the next household. Due to logistical challenges during the COVID-19 pandemic, no mosquitoes were collected in January, March and April of 2021 in Ribangua, and in April 2021 in Palmeira.

### 2.3. Data Sources, Quality Control and Analytical Approach

#### 2.3.1. Mosquito Host-Seeking Data (Quality Control and Analysis)

Additional information was gathered using a questionnaire (ODK_Collect v1.4.4). Questions were answered after each sampling period and for each household to assess the following: (i) whether the volunteer left the tent during the trapping period, other than for a short bathroom break (yes/no), (ii) if the CDC Miniature Light Trap was still operational after a trapping period (fan and light still on, both yes/no), (iii) if the Collection Bottle Rotator rotated properly (rotator position at time of collection), (iv) if there were ants present in the collection cups (cup number(s) with ants), (v) if people cooked inside the room during the trapping period (yes/no), (vi) if vector control tools were used, e.g., topical repellents, repelling coils (yes/no, and which ones if answered ‘yes’) and (vii) if and how many people slept in the tent, room and house the previous night (number per location).

For Ribangua, data were included from 62 collection events indoors and 61 outdoors during the daytime period, and 62 collection events indoors and 59 outdoors during the nighttime period. For Palmeira, data were included from 26 collection events for each location (indoors and outdoors) and period (daytime and nighttime).

The number of mosquito bites per person per night (bpn) for each species was calculated using collection date, trap position (indoor or outdoor), and species as categorical variables. The numerical variables were the number of mosquitoes found in each cup. We assumed that each mosquito collected in a collection cup is equivalent to a human bite. All data analyses and visualizations were performed using R version 4.3.2. [30].

#### 2.3.2. Sunrise and Sunset Data (Sources and Processing)

Local time for sunrise and sunset in Manhiça District and other locations in Africa for 2021 (i.e., 365 days) were analyzed using data from the National Oceanic and Atmospheric Administration (NOAA) Global Monitoring Laboratory [31]. Sunrise is defined as the time the geometric center of the sun reaches the horizon in the morning, while sunset was when it crosses below the horizon in the evening [32]. Locations across Africa were selected from various longitudes (−10° [Guinea] to 50° [Somalia], in 10° increments) at latitude 10°, and from various latitudes (−30° [South Africa] to 30° [Libya] in 10° increments) at a longitude of 20°. These datasets were processed to generate annual sunrise–sunset curves and to compare daylight cycle variability within and across regions. Note that the local times for sunrise and sunset provided in this paper are for those exact coordinates, and that sunrise and sunset times can vary throughout a single country, depending on the exact latitude and longitude coordinates.

## 3. Results

### 3.1. Mosquito Time of Biting in Manhiça District

A total of 151 anopheline mosquitoes were collected from the two sentinel areas, including *An. tenebrosus* (83.4%), *An. funestus* s.l. (6.6%), *An. ziemanni* (4.0%), *An. gambiae* s.l. (3.3%), *An. squamosus* (0.7%) and 3 specimens (2.0%) that could not be identified. Overall, 81.5% were collected outdoors.

**Figure 1:**
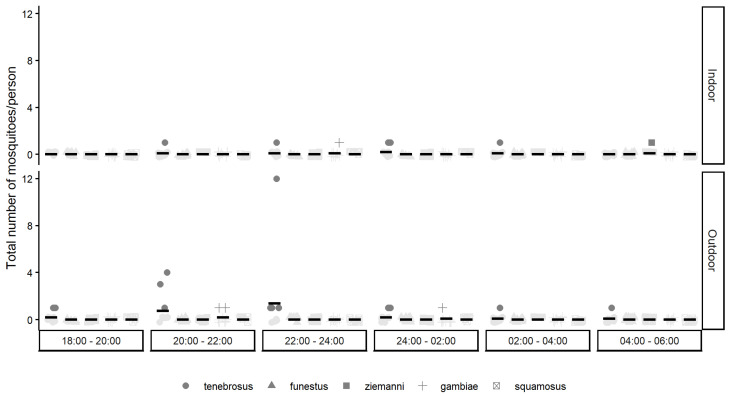
The number of mosquitoes per person per night (*y*-axis) collected for each anopheline mosquito species in Ribangua over time (every 2 h) (*x*-axis), both indoors (top panel) and outdoors (bottom panel).

In Ribangua, a total of 41 mosquitoes were collected, all during the nighttime hours (i.e., between 18:00 and 6:00). A total of eight mosquitoes were collected indoors during five trap nights, and included five *An. tenebrosus*, one *An. gambiae* s.l., one *An. ziemanni* and one unidentified specimen. A total of 33 mosquitoes were collected outdoors during 17 trap nights. *An. tenebrosus* (87.9%) was the most abundant species, followed by *An. gambiae* s.l. (9.1%) and one unidentified specimen (3%). Peak *An. tenebrosus* abundance, expressed as the number of bites per person per night (bpn), was recorded in September 2020 (indoors) and November 2020 (outdoors) (Figure S2), but note again that three months of data collection during the rainy season are missing. The outdoor peak biting time of *An. tenebrosus* was between 22:00 and 24:00 (Figure 1). *An. gambiae* s.l. and *An. ziemanni* were caught indoors during the periods of 22:00–24:00 and 04:00–06:00, respectively. The *An. gambiae* s.l. outdoor specimens were collected between the hours of 20:00–22:00 h and 24:00–02:00 (Figure 1).

**Figure 2:**
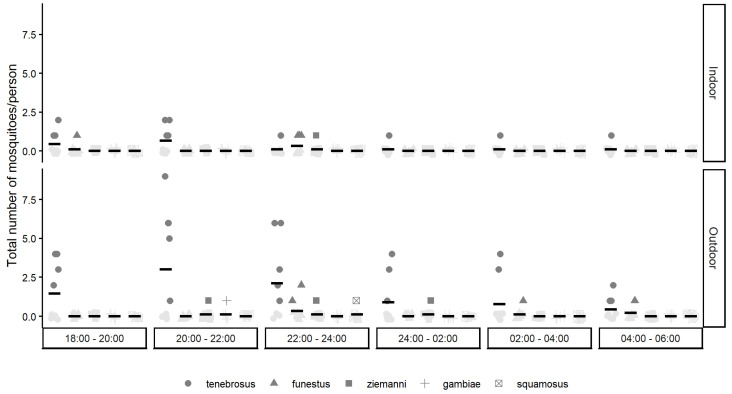
The number of mosquitoes per person per night (*y*-axis) collected for each anopheline mosquito species in Palmeira over time (every 2 h) (*x*-axis), both indoors (top panel) and outdoors (bottom panel).

In Palmeira, albeit a much shorter collection period (four months), a larger number of mosquitoes were collected (n = 110, Figure S3). All but one mosquito was collected during the nighttime hours (98.2%). A total of 20 mosquitoes were collected indoors over 9 trap nights. *An. tenebrosus* (70%) was the most abundant species, followed by *An. funestus* s.l. (20%), *An. ziemanni* (5%) and one unidentified specimen (5%). A total of 89 mosquitoes were collected outdoors during 10 trap nights. *An. tenebrosus* (87.6%) was the most abundant species, followed by *An. funestus* s.l. (6.7%), *An. ziemanni* (3.4%), *An. gambiae* s.l. (1.1%) and *An. squamosus* (1.1%). The highest number of bpn for *An. tenebrosus* was recorded in June 2021 (both indoors and outdoors) (Figure 2). Note again that data from a large part of the rainy season are missing. The peak biting time of *An. tenebrosus* outdoors was during 20:00–22:00. *An. funestus* s.l. (the second most abundant species) had its highest bpn outdoors between 22:00 and 24:00 (Figure 2). The *An. ziemanni* (n = 1) collected outdoors during the daytime period (i.e., between 6:00 and 18:00) was collected during the hours of 16:00–18:00 (note that Figure 2 only shows mosquitoes that were collected between 18:00 and 6:00).

### 3.2. Sunrise and Sunset Patterns in Manhiça District and Across Africa

**Figure 3:**
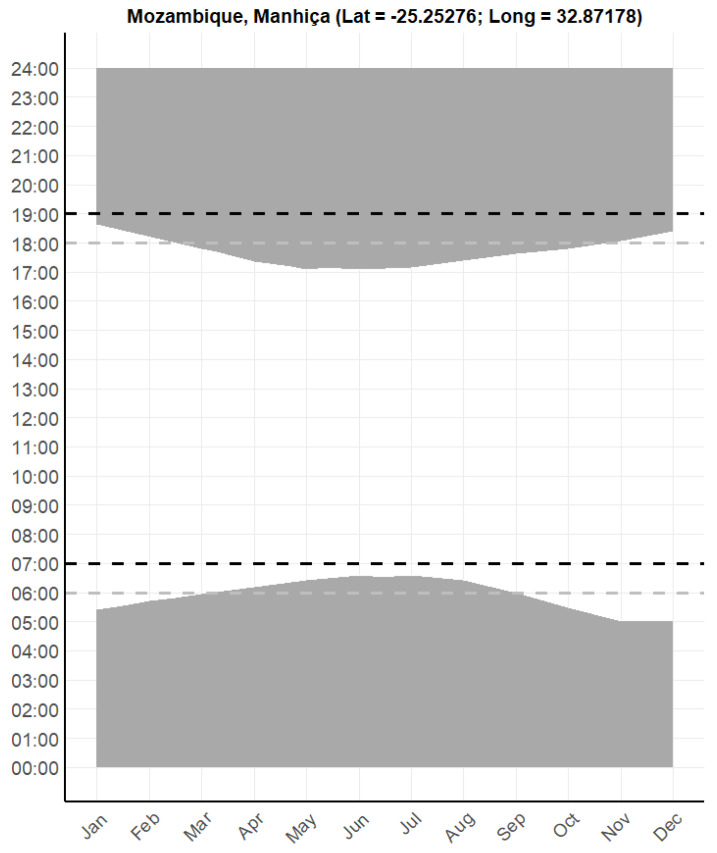
Sunset and sunrise patterns in Manhiça district, Mozambique. The *y*-axis represents local time (0–24 h), and the *x*-axis represents the months of the year 2021. The gray area visualizes nighttime, the dotted lines indicate typical start (18:00 in gray; 19:00 in black) and end (06:00 in gray; 07:00 in black) times in malaria mosquito entomological surveillance programs. Data from the NOAA Global Monitoring Laboratory (https://gml.noaa.gov/grad/solcalc/, accessed on 2 September 2024).

In 2021, the sunrise in Manhiça occurred between 04:50 (period 24 November to 7 December) and 06:35 (29 June to 7 July) local time, and sunset between 17:06 (31 May to 16 June) and 18:44 (7–17 January) (Figure 3). During the early months of the study (November and December 2020), the region experienced longer daylight hours, with sunrise as early as 04:50 h and sunset as late as 18:42 h. By the end of the study period in July 2021, daylight hours had shortened, with sunrise as late as 06:27 and sunset as early as 17:23. The *An. ziemanni* specimen that was collected during daytime (defined in this study as the period from 06:00 h to 18:00) was collected between 16:00 and 18:00. Since sunset occurred at 17:06 on that particular day, this specimen could thus have been collected either before (i.e., between 16:00 and 17:06 h) or after sunset (i.e., between 17:06 and 18:00 h), which means we cannot label this mosquito as a daytime or nighttime biter without more detailed investigations.

**Figure 4:**
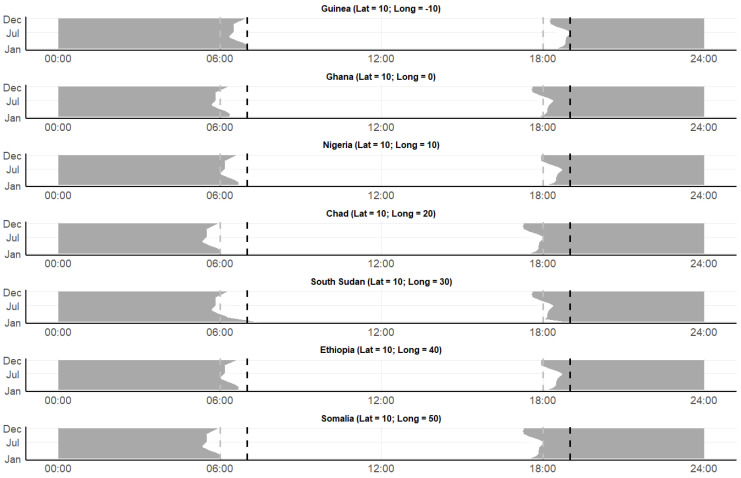
Daylight cycles, sunset and sunrise patterns in locations across Africa from various longitudes (−10° [Guinea] to 50° [Somalia], in 10° increments) at latitude 10°. The *x*-axis represents time (0–24 h), and the *y*-axis represents the months of the year 2021. The gray area visualizes nighttime, the dotted lines indicate typical start (18:00 in gray; 19:00 in black) and end (06:00 in gray; 07:00 in black) times in malaria mosquito entomological surveillance programs. Data from the NOAA Global Monitoring Laboratory (https://gml.noaa.gov/grad/solcalc/, accessed on 2 September 2024).

Across Africa, sunrise and sunset patterns vary with latitude and longitude. In general, moving across longitude at latitude 10° (i.e., parallel to the equator), both sunrise and sunset times differ by less than an hour throughout the year (Figure 4). For example, sunrise times vary between 05:38 and 06:23, and sunset times between 17:35 and 18:25 in Ghana, with values of 05:18–06:03 and 17:15–18:06 being recorded in Somalia, respectively.

**Figure 5:**
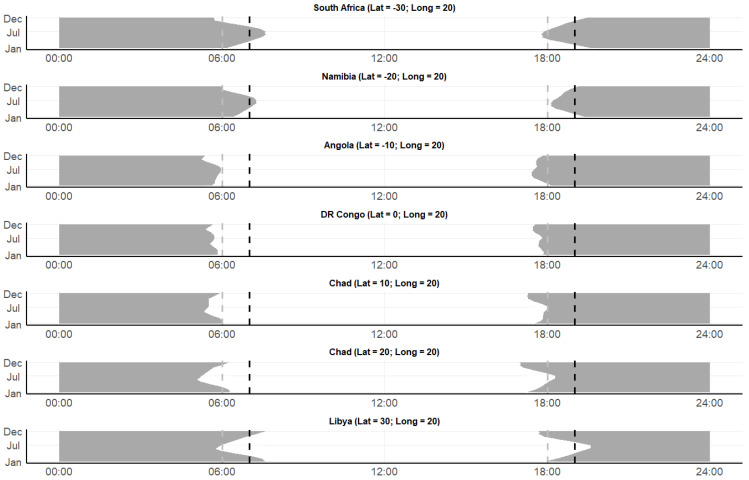
Daylight cycles, sunset and sunrise patterns in locations across Africa from various latitudes (−30° [South Africa] to 30° [Libya] in 10° increments) at a longitude of 20°. The *x*-axis represents time (0–24 h), and the *y*-axis represents the months of the year 2021. The gray area visualizes nighttime, the dotted lines indicate typical start (18:00 in gray; 19:00 in black) and end (06:00 in gray; 07:00 in black) times in malaria mosquito entomological surveillance programs. Data from the NOAA Global Monitoring Laboratory (https://gml.noaa.gov/grad/solcalc/, accessed on 2 September 2024). Note that two geographic locations are both within Chad.

Moving across latitude at a longitude of 20° (i.e., perpendicular to the equator), the differences in sunrise and sunset local times across a full year are smaller near the equator (DR Congo: 05:20–05:51, and 17:27–17:58, respectively) but become larger when you move north (Libya: 05:38–07:37, and 17:40–19:45, respectively) or south (South Africa: 05:31–07:37, and 17:47–19:45, respectively) (Figure 5).

## 4. Discussion

*Anopheles tenebrosus*, *An. funestus* s.l., *An. gambiae* s.l., *An. squamosus* and *An. ziemanni* were present in southern Mozambique, with *An. tenebrosus* being the most abundant species. Most mosquitoes (81.5%) were collected outdoors, and all mosquitoes were captured during the nighttime period, with the exception of a single *An. ziemanni* (0.7% of all mosquitoes caught) that was collected during the daytime (but see our discussion below).

*An. tenebrosus* exhibited both endophilic and exophilic behaviors, a phenomenon that has recently been demonstrated elsewhere in Mozambique [28]. More importantly, *An. tenebrosus* has also been incriminated as a malaria vector in this area [28,33]. Historically, the main malaria vectors in southern Mozambique were *An. funestus* s.s. and, to a lesser extent, *An. arabiensis* [34,35,36,37]. The fact that *An. funestus* s.l. only comprised a small part of our mosquito population is not surprising as the expansion and intensity of IRS campaigns in the area over the years are known to quickly reduce *An. funestus* abundance [18,28]. It is therefore important to continuously monitor species like *An. tenebrosus* in pre-elimination areas, since their different feeding and resting behaviors [38,39] can make ITNs and/or IRS less effective.

There are several limitations to this study. First, the mosquito numbers were low compared to the studies performed in Senegal [20], Central African Republic [19] and Kenya [21]. Although mosquito abundance is expected to be lower in pre-elimination areas that see a high insecticidal pressure on mosquito populations [40,41], the small sample sizes in this study may not allow us to detect biting behaviors after dawn and before dusk. Second, due to logistical challenges because of the COVID-19 pandemic, mosquito collections were not performed monthly during the malaria transmission season. Therefore, it cannot be ruled out that daytime biting did not occur during those months. Third, the study was not conducted in both sentinel areas for the same amount of time (surveillance in Palmeira started later). Fourth, while the HBTTs capture anophelines mosquitoes, there remains the inability to directly compare this surveillance tool with human landing catches, which are still considered the gold standard in vector surveillance. Future studies should evaluate the abundance, diversity and behaviors of mosquito species in different geographical locations across seasons.

The fifth limitation is that we pre-defined the daytime period as the period between 06:00 and 18:00, and the nighttime period as the period between 18:00 and 06:00. However, we noticed that when we initiated our surveillance at 06:00, the sun had frequently risen already, and vice versa at 18:00. This mismatch between our daytime/nighttime definitions and reality creates a surveillance framework that cannot fully capture the actual daylight cycles, which could lead to an under- or overestimation of mosquito activity during day- or nighttime. While adjusting surveillance windows to match the precise daily and seasonal shifts in sunrise and sunset would be ideal, we acknowledge that doing so is often operationally challenging. Field teams typically work with standardized trapping schedules for logistical, financial and safety reasons, making continuous adjustment impractical. A balance is therefore needed between scientific accuracy and field feasibility. One potential solution is the use of hourly rotating collection systems or other automated traps, which can capture fine-scale temporal patterns without requiring constant manual readjustment of field schedules.

Our approach is, however, not unusual. It is common in entomological surveillance studies to assess malaria mosquito abundance, diversity and/or behaviors from either 18:00 to 06:00, or 19:00 to 07:00 (e.g., [22,23,24,25,42,43,44]), as malaria mosquitoes are typically active after sunset and before sunrise. However, according to the NOAA solar data, the actual start of sunrise and sunset in Manhiça district (Palmeira location) during this study period varied between 04:50 and 06:35, and 17:06 and 18:43, respectively. This means that there was a period when (a) nighttime collections started when it was still light outside, and ended when the sun was up, and (b) daytime collections started when it was still dark outside and ended when the sun was already set. This also means that the *An. ziemanni* that was collected outdoors between 16:00 and 18:00—and that we considered a daytime biter—might have been caught after sunset (17:06 that specific day) and could thus very well be a nighttime feeder. Similarly, two *An. tenebrosus* collected between 04:00 and 06:00 in Palmeira were captured on a morning when sunrise occurred at 05:44, meaning that a fraction of this trapping interval also took place after sunrise. Although the likelihood of true diurnal activity during this short interval is low, these cases further illustrate how fixed surveillance windows can complicate the interpretation of biting behavior relative to actual light conditions. We argue that this is a limitation in entomological surveillance studies in general, with differences in the time of sunrise and sunset changing across longitude and latitude through the year. Distinguishing whether observed biting patterns reflect true behavioral shifts or are simply influenced by changing daylight conditions is inherently challenging. Seasonal changes in daylength, shifting sunrise and sunset times, and the potential behavioral effects of sustained vector control pressure [45,46] may all modify the apparent timing of host-seeking. Our study was not designed to disentangle these drivers, and more detailed longitudinal data (capturing fine-scale temporal changes in both mosquito activity and environmental light conditions) would be required to do so.

Overall, our data suggests that daytime biting may not be a threat for malaria elimination in southern Mozambique. However, continuous monitoring during daytime may be necessary to adequately assess the efficacy of current vector control tools, and see if vector control efforts lead to a shift in mosquito behaviors to, e.g., more daytime biting mosquitoes. However, we acknowledge that it may be more important to match mosquito activity with human activity patterns directly, regardless of whether it is dark or light outside, as this interaction throughout the day will determine the effectiveness of vector control tools such as ITNs [11,47,48]. This requires monitoring important human behaviors (such as time to go to bed and total sleep time), which are known to change with seasons, and thus daylight cycles [49], although other factors, such as changes in temperature, social (e.g., cultural norms) and individual attributes (e.g., age, gender, exercise) may play a more important role [50]. If human behaviors cannot be monitored, we suggest that entomological programs distinguish between daytime and nighttime biting mosquitoes while considering actual daylight cycles to more accurately determine the current gaps in protection [51].

## Data Availability

The original contributions presented in this study are included in the article/Supplementary Material. Further inquiries can be directed to the corresponding author.

## Supplementary Materials

The following supporting information can be downloaded at: https://www.mdpi.com/article/10.3390/insects16121264/s1, Figure S1: Map showing study sites in Maputo province, southern Mozambique. Figure S2: The number of mosquitoes (*y*-axis) collected for each anopheline mosquito species in Ribangua over time (monthly) (*x*-axis), both indoors (top panel) and outdoors (bottom panel). The “n” value indicates the number of collection periods. Note that data for January, March and April 2021 are missing due to logistical challenges during the COVID-19 pandemic. Figure S3: The number of mosquitoes (*y*-axis) collected for each anopheline mosquito species in Palmeira over time (monthly) (*x*-axis), both indoors (top panel) and outdoors (bottom panel). The “n” value indicates the number of collection periods. Note that data for April 2021 are missing due to logistical challenges during the COVID-19 pandemic. Excel S1: Datafile with mosquito host-seeking activity times for Ribangua. Excel S2: Datafile with monthly mosquito abundance for Ribangua. Excel S3: Datafile with mosquito host-seeking activity times for Palmeira. Excel S4: Datafile with monthly mosquito abundance for Palmeira.
