# Supplementary figures and images for: Malaria Mosquito Host-Seeking Activity Times in Manhiça District, Rural Mozambique, and the Need to Better Match Entomological Surveillance Strategies to Daylight Cycles
Source: Insects. 2025 Dec 12;16(12):1264. doi: 10.3390/insects16121264 (PMC12734131; doi:10.3390/insects16121264)

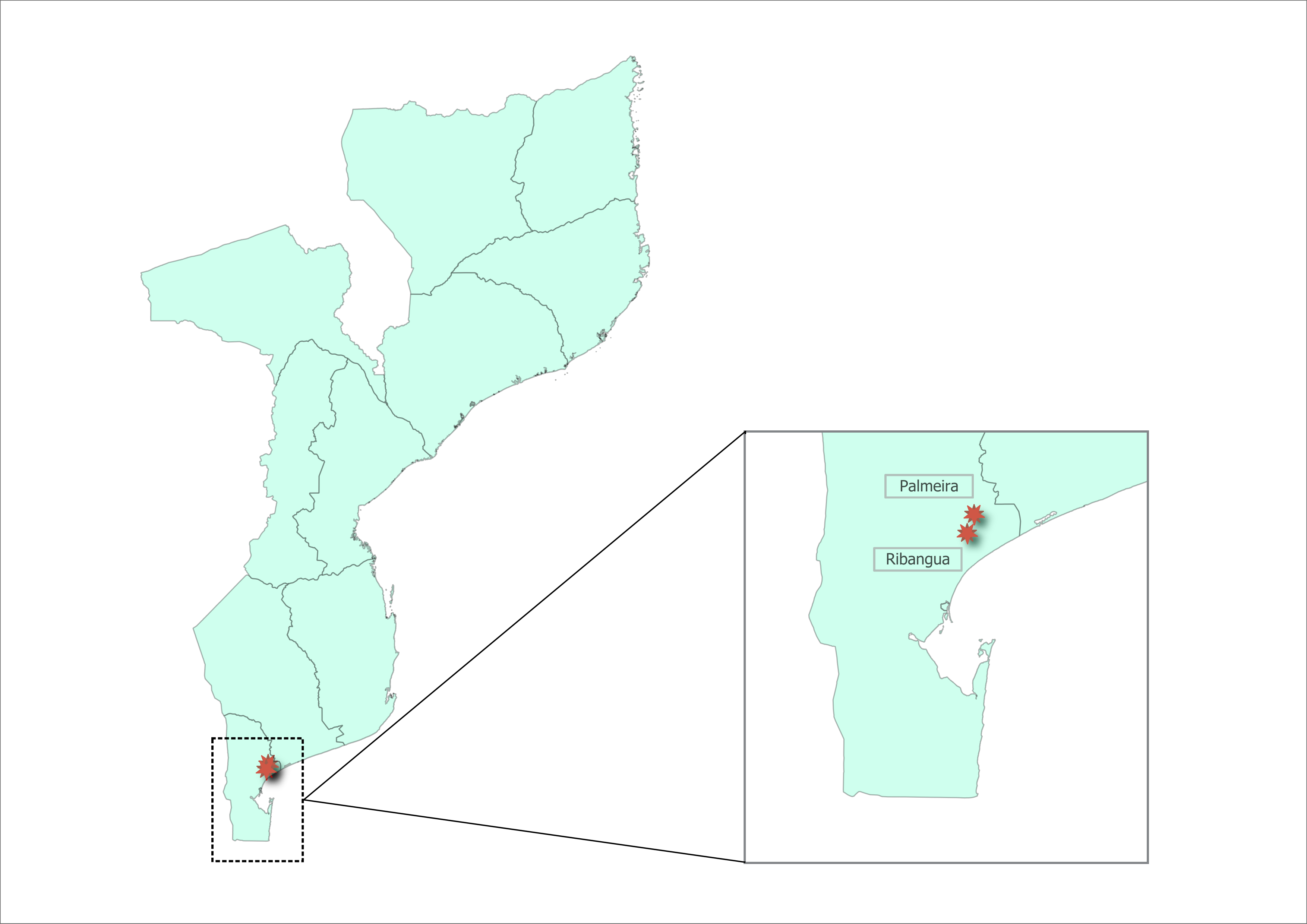

Supplement: Supplementary file 1 [file insects-16-01264-s001.zip › SI_Jobe_Communication_Figure_S1.png]

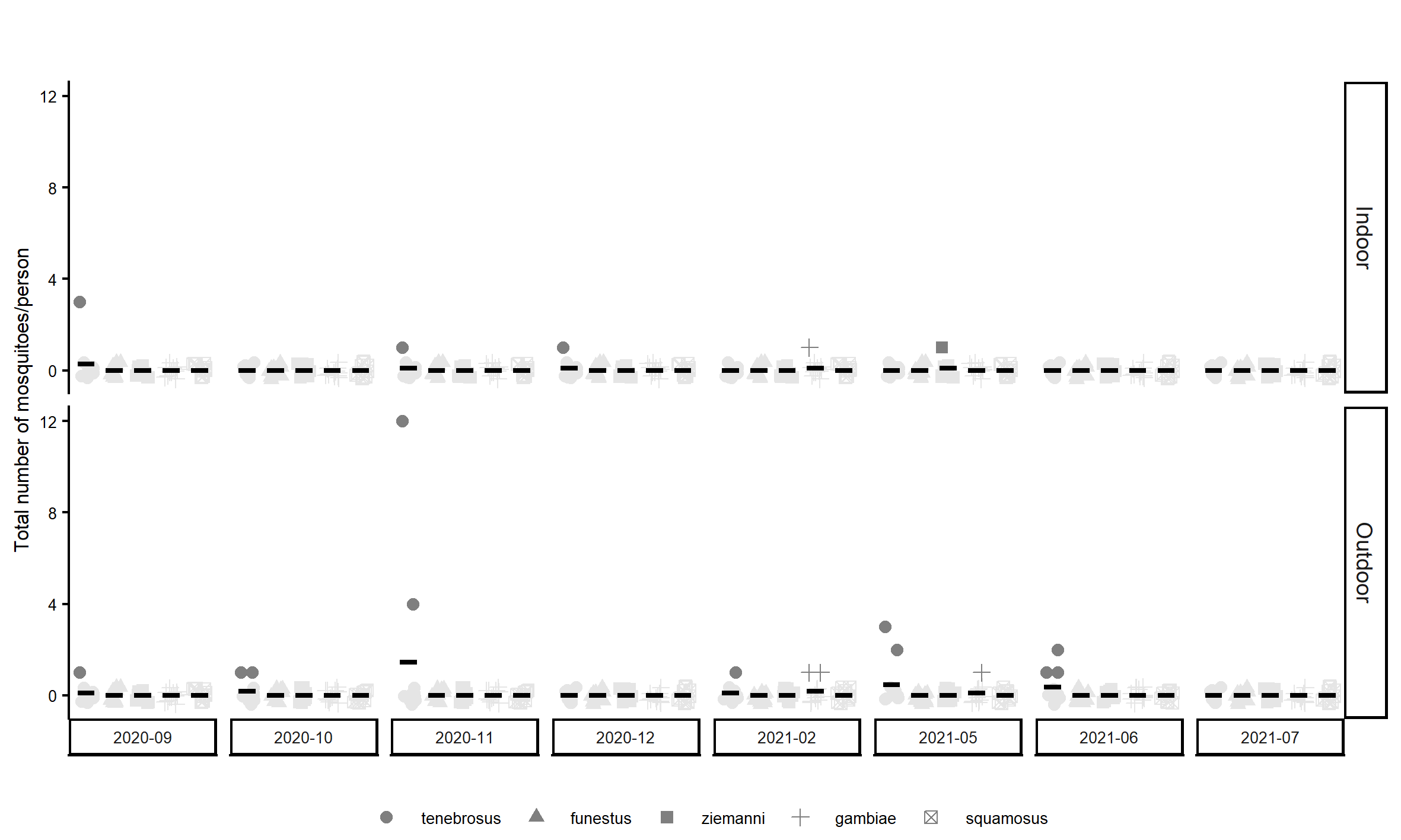

Supplement: Supplementary file 1 [file insects-16-01264-s001.zip › SI_Jobe_Communication_Figure_S2.png]

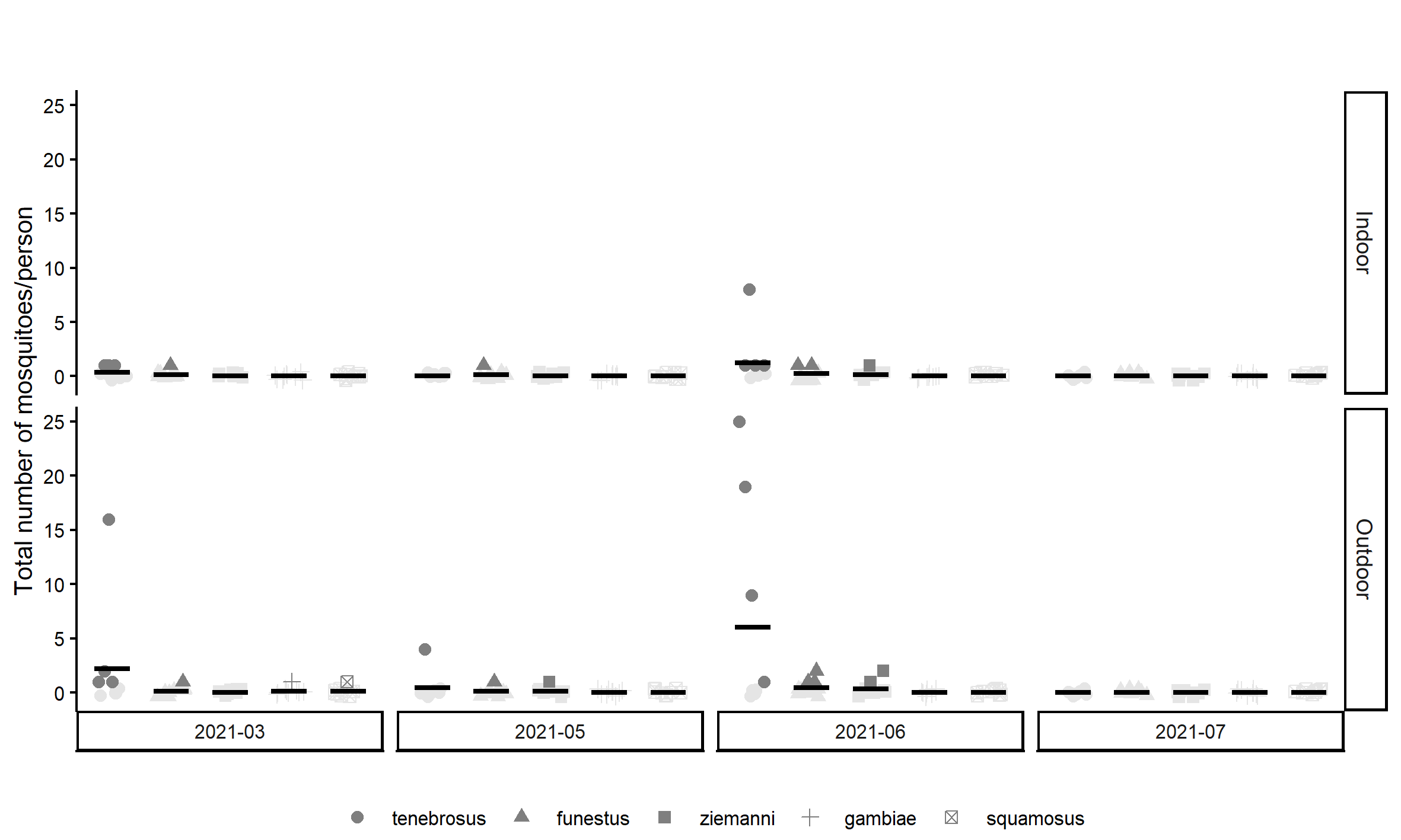

Supplement: Supplementary file 1 [file insects-16-01264-s001.zip › SI_Jobe_Communication_Figure_S3.png]
